# What's Missing from Data Modernization? A Focus on Structural Racism

**DOI:** 10.1089/heq.2023.0086

**Published:** 2023-10-17

**Authors:** Jamila M. Porter, Brian C. Castrucci, Jacquelynn Y. Orr

**Affiliations:** ^1^ Office of the CEO, de Beaumont Foundation, Bethesda, Maryland, USA.; ^2^ Health Program, The Kresge Foundation, Troy, Michigan, USA.

**Keywords:** data modernization, structural racism, data justice, community power, public health, health equity

## Abstract

Public health data modernization efforts frequently overlook the far-reaching effects of structural racism across the data life cycle. Modernizing data requires creating data ecosystems grounded in six principles: dismantling structural racism and building community power explicitly; centering justice in all stages of data collection and analysis; ensuring communities can govern their data; driving positive population-level change; engaging nonprofit organizations; and obtaining commitments from governments to make changes in policy and practice. As government agencies spearhead and finance data modernization initiatives, it is imperative that they address structural racism head-on and integrate these principles into all aspects of their work.

The COVID-19 pandemic yielded many painful lessons for our nation. One critical lesson we learned is that the data systems that undergird our understanding of health in the United States are systemically flawed. These antiquated, disjointed, and chronically underfunded data systems contained “gross gaps in data for race and ethnicity for COVID-19 cases and deaths”^1^ and denied agencies and communities critical information that could have saved lives. Although the decades-long need to modernize our public health data systems has finally become a priority, government-funded data modernization initiatives have erroneously framed this as a technical problem. This framing ignores a fundamental system of power that impacts every aspect of the data life cycle: structural racism. Given that structural racism is a root cause of health inequities,^2^ data modernization efforts must be centered around *justice*—shifting power and resources to communities that have been systematically oppressed.

Racial health inequities are complex, pervasive, and deeply entrenched. These inequities are not accidental, inevitable, behavioral, or biological; they have been intentionally and systematically created over centuries by policies, practices, and decisions. Data are often exalted as objective truth, seemingly reflective of unequivocal aspects of human existence. However, data systems are neither inherently objective nor neutral; rather, they are constructed—and thus biased—by the people, beliefs, and interests that drive their creation. When these data are used in isolation, and without the governance of the communities they impact and reflect, at best, they can create a distorted view of a community's health and well-being; at worst, they can fuel narratives, policies, and decisions that perpetuate structural racism and the racial health inequities it creates.

Data systems used to track drivers of community health—such as vital statistics,^3^ census data,^4^ housing data,^5^ and standardized testing data^6^—are rooted in racist systems and assumptions. As a result, they consistently fail to measure structural racism as a primary driver of these inequities. While data are powerful tools that can be used to measure multiple dimensions of community health, they are also commonly and spuriously used to document racial health inequities as if these inequities are innate. “Race” is a social construct—an arbitrary categorization and ever-changing social group assignment that lacks scientific or biological meaning.^7^ However, *racism* and its adverse impacts are real. The use of arbitrary racial categorizations has created a rigid racial caste system that continues to levy quantifiably deleterious consequences on communities of color across the lifespan—from adverse birth outcomes to lower life expectancies.^2,8^ Structural racism, “whether through force, deprivation, or discrimination,”^9^ is a fundamental cause of disability, disease, and death.

In April 2021, the Centers for Disease Control and Prevention (CDC) declared racism a serious public health threat that directly affects the well-being of millions of Americans.^10^ This declaration must explicitly extend to CDC's data modernization efforts.^11^ Anti-racism, equity, justice, and community power must be at the center of any effort to modernize our data systems, including how they are created and operate, what they measure, and whom they benefit. To realize their full potential, data systems must be modernized to become *ecosystems*—interconnected generators of information that are linked across multiple sectors and agencies, wherein the needs and voices of communities most impacted by structural racism are prioritized. The following six principles must guide our efforts to create truly modernized data ecosystems:

1. **Dismantling structural racism and building community power must be explicit and intentional.**
  An equity- and justice-focused local data ecosystem must center anti-racism, equity, justice, and community power. This means that the information generated by the data ecosystem uplifts community voices, reflects their lived experiences, and places their collective goals at the forefront. An ecosystem's focus on these principles cannot be implied—it must be intentional, explicit, and unequivocal.
2. **Data connectivity and integration across sectors must center justice.**
  Previous efforts to modernize public health data have focused on improving data capabilities and connecting data across sectors, such as between clinical care and social services. However, principles of equity and social justice—both of which are critical to eliminate racial health inequities—have not been consistently centralized in efforts to modernize data. What we measure, how we measure it, and the problems we seek to address through the data we collect must continuously seek to upend structural racism and advance health equity goals.
3. **Data must be defined and governed by the people who generate it.**
  Differences in health outcomes between racial groups—particularly between white, Black, and Indigenous people—have “been part of the American landscape for 400 years.”^12^ Structural racism drove these differences in health and shaped the data systems we've used to document these differences over time. Therefore, it is critical that communities—particularly those that have been oppressed by structural racism—have the right to govern their data. The data of Indigenous people and Tribal nations are often defined, owned, and controlled by state and federal agencies, rather than the Tribes themselves.^13^ This results in a dual disparity: Tribal data are simultaneously inaccurate and misrepresentative, while also difficult (if not impossible) to access. Communities—particularly communities of color—have a right to determine and govern how data about them are collected, used, applied, and shared. Modernized data ecosystems must be responsible and ethical, provide authority of control to communities, and be designed for collective benefit (wherein entire communities, and not just contributing individuals, benefit from the application and use of data).^14^
4. **Community-level data must be used to drive positive population-level change.**
  Data modernization efforts have often focused on individualized clinical data, such as health care services and vaccinations. While important, these data generally focus on downstream services and individual outcomes, such as coordination of care, referrals, and client services. Modernizing data requires us to focus *upstream*—preventing adverse health and social outcomes at the population level before they occur. This can be accomplished by connecting community-level data systems across sectors, including data related to income and wealth, land use and planning, public health, housing, education, civic engagement, and transportation and public transit. By connecting multiple community-level variables, local data ecosystems will be better equipped to inform broad, community-level decisions that have the potential to change entire trajectories of population health.
5. **Nonprofit organizations are key to building bridges to improve data.**
  Due to our nation's long legacy of structural racism and state-sanctioned violence, communities of color have understandably been mistrustful of government.^12,15^ However, nonprofit organizations—especially those with deep roots in their communities—can serve as trusted third parties that work closely with and build bridges between government agencies and communities of color. Nonprofit organizations, especially those engaged in community power building efforts (e.g., community organizing, leading grassroots advocacy campaigns), also bring many other strengths to this work: They often have strong community connections, organizational missions, and strategic priorities that focus on shifting power. These characteristics are essential to build data ecosystems that center the needs and voices of marginalized communities.
6. **Government commitments to change policies and practices are essential.**
  Government agencies often create and maintain data systems that are used to track measures of community health. However, governments have also perpetuated structural racism through unjust public and organizational policies, inequitable budget allocations, and other discriminatory decision-making processes and practices. For all these reasons, government agencies are critical to any fundamental systems change. Databases, data systems, and research studies that have been funded by federal, state, and local governments have been paid for by taxpayers. Hence, these data should be readily available to and accessible by the public. Given the immense power they wield, governments have a responsibility to make and honor explicit commitments to change their policies, practices, and operations.^16^ They must make data openly available to the very members of the public who fund these data systems and who are reflected in them. Governments must also build and utilize collaborative governance processes that change existing power structures and ensure communities of color are full and formal partners in data-related decision-making.^17^

Progress toward our public health and social justice goals demands a complete transformation of how data are gathered, analyzed, understood, and applied. This transformation must go beyond mere technical endeavors to link and disaggregate data, bolster capabilities, or refine integration and interoperability. It demands deliberate, values-driven decisions that place anti-racism, equity, justice, and community power building at the core of data modernization efforts.

We must do more than just talk; we must take action to drive change, as that is where “the hard operational work begins.”^18^ The ultimate goal of data modernization should be to shift power, rectify injustices against communities of color, and ensure communities have the resources and autonomy necessary to advance their own agendas.

## Abbreviation Used

CDC: Centers for Disease Control and Prevention
